# Gastrointestinal disorders in Curry–Jones syndrome: Clinical and molecular insights from an affected newborn

**DOI:** 10.1002/ajmg.a.38232

**Published:** 2017-04-06

**Authors:** Kristen Wigby, Stephen R. F. Twigg, Ryan Broderick, Katherine P. Davenport, Andrew O. M. Wilkie, Stephen W. Bickler, Marilyn C. Jones

**Affiliations:** ^1^ Department of Pediatrics University of California San Diego and Rady Children's Hospital ‐ San Diego San Diego California; ^2^ Clinical Genetics Group Weatherall Institute of Molecular Medicine University of Oxford Oxford UK; ^3^ Department of Surgery University of California San Diego and Rady Children's Hospital ‐ San Diego San Diego California

**Keywords:** Curry–Jones syndrome, gastrointestinal smooth muscle hamartomas, *SMO* somatic mosaic mutations

## Abstract

Curry–Jones syndrome (CJS) is a pattern of malformation that includes craniosynostosis, pre‐axial polysyndactyly, agenesis of the corpus callosum, cutaneous and gastrointestinal abnormalities. A recurrent, mosaic mutation of *SMO* (c.1234 C>T; p.Leu412Phe) causes CJS. This report describes the gastrointestinal and surgical findings in a baby with CJS who presented with abdominal obstruction and reviews the spectrum of gastrointestinal malformations in this rare disorder. A 41‐week, 4,165 g, female presented with craniosynostosis, pre‐axial polysyndactyly, and cutaneous findings consistent with a clinical diagnosis of CJS. The infant developed abdominal distension beginning on the second day of life. Surgical exploration revealed an intestinal malrotation for which she underwent a Ladd procedure. Multiple small nodules were found on the surface of the small and large bowel in addition to an apparent intestinal duplication that seemed to originate posterior to the pancreas. Histopathology of serosal nodules revealed bundles of smooth muscle with associated ganglion cells. Molecular analysis demonstrated the *SMO* c.1234 C>T mutation in varying amounts in affected skin (up to 35%) and intestinal hamartoma (26%). Gastrointestinal features including structural malformations, motility disorders, and upper GI bleeding are major causes of morbidity in CJS. Smooth muscle hamartomas are a recognized feature of children with CJS typically presenting with abdominal obstruction requiring surgical intervention. A somatic mutation in *SMO* likely accounts for the structural malformations and predisposition to form bowel hamartomas and myofibromas. The mutation burden in the involved tissues likely accounts for the variable manifestations.

## 1 INTRODUCTION

Curry–Jones syndrome (CJS; OMIM #601707) is a pattern of malformation that includes craniosynostosis, pre‐axial polysyndactyly, agenesis of the corpus callosum, cutaneous and gastrointestinal abnormalities (Temple et al., 1995). The first two cases were presented as unknown syndromes at the David W. Smith workshop on malformations in 1987 (Curry, 1987). Cohen (1988) recognized the cases as a pattern of malformation and termed the CJS after the physicians who had described the first two cases. Eleven unrelated cases have been reported in the literature presenting with craniofacial abnormalities, patchy cutaneous findings, and polysyndactyly (Grange et al., 2008; Mingarelli, Mokini, Castriota‐Scanderbeg, & Dallapiccola, 19999; Temple et al., 1995; Thomas et al., 2006; Twigg et al., 2016).

Until recently, the genetic etiology of CJS was not known. All cases of CJS occurred sporadically and karyotype analyses of blood and fibroblasts from affected skin were normal. Based on clinical observations including asymmetric features and patchy cutaneous manifestation, it was hypothesized that CJS was due to a somatic mutation arising in early post‐zygotic embryonic development. However, symmetric polydactyly and syndactyly was not consistent with this hypothesis. Grange et al. (2008) reported two new children with CJS including one child with a desmoplastic medulloblastoma and another child with a trichoblastoma of the skin and thus hypothesized that CJS may be due to a defect in the sonic hedgehog (Shh) pathway. However, sequencing of *PTCH* and *GLI*, genes coding for key proteins in the Shh signaling pathway, was negative. Recently, Twigg et al. (2016) identified by whole exome sequencing of affected skin, a somatic heterozygous variant in *SMO* (c.1234C>T; p.Leu412Phe). This gene encodes the G‐protein coupled receptor smoothened which is involved in the HH‐GLI signaling pathway. The authors then identified varying amounts of the identical mutant allele by deep sequencing of affected tissues in eight unrelated individuals with CJS, providing additional evidence for the molecular cause of CJS.

Gastrointestinal abnormalities are a cardinal feature of CJS. Previously described findings include pseudo‐obstruction, malrotation, and gastrointestinal myofibromas or smooth muscle hamartomas (Grange et al., 2008; Temple et al., 1995). Here, we present a child with CJS who presented with abdominal distension in the newborn period. This case, listed in table 1 of Twigg et al. (2016) as case #10, was found to have the recurrent, mosaic mutation of *SMO* (c.1234 C>T; p.Leu412Phe) that causes CJS. This report presents a detailed clinical description and is accompanied with a more complete molecular analysis. In addition we compare the gastrointestinal features with prior cases in the literature and discuss the developmental pathophysiologic correlates to further delineate the syndrome.

## 2 CASE DESCRIPTION

The proband is a female infant born at 41 weeks EGA to an 18‐year‐old G1P0 Hispanic‐Samoan mother and non‐consanguineous 18‐year‐old Hispanic father. The mother had routine prenatal care and there were no significant exposures. Pregnancy was complicated by pyelectasis and absent cavum septum pellucidum detected on ultrasound during the second trimester fetal anatomy survey. Upon transfer of care at 35 weeks (after the family relocated), she was started on weekly non‐stress tests due to the presence of suspected fetal anomalies. A repeat ultrasound obtained at 39 3/7 weeks GA showed accelerated fetal growth (gestational age equivalents: biparietal diameter: 40 0/7 weeks, head circumference 40 6/7 weeks, abdominal circumference 39 6/7 weeks, femur length 37 1/7 weeks), and apparent fusion of the anterior lateral ventricles, concerning for alobar holoprosencephaly. The infant was delivered by cesarean section after a failed induction of labor with Apgars of 9 and 9 at 1 and 5 min, respectively. The infant was large for gestational age with a birth weight of 4,165 g (96th percentile, *z*‐score 1.88), length of 53.3 cm (98th percentile, *z*‐score 2.23), and occipitofrontal head circumference of 38.5 cm (∼100th percentile, *z*‐score 3.90) (WHO 0–2 years female growth chart).

**Figure 1:**
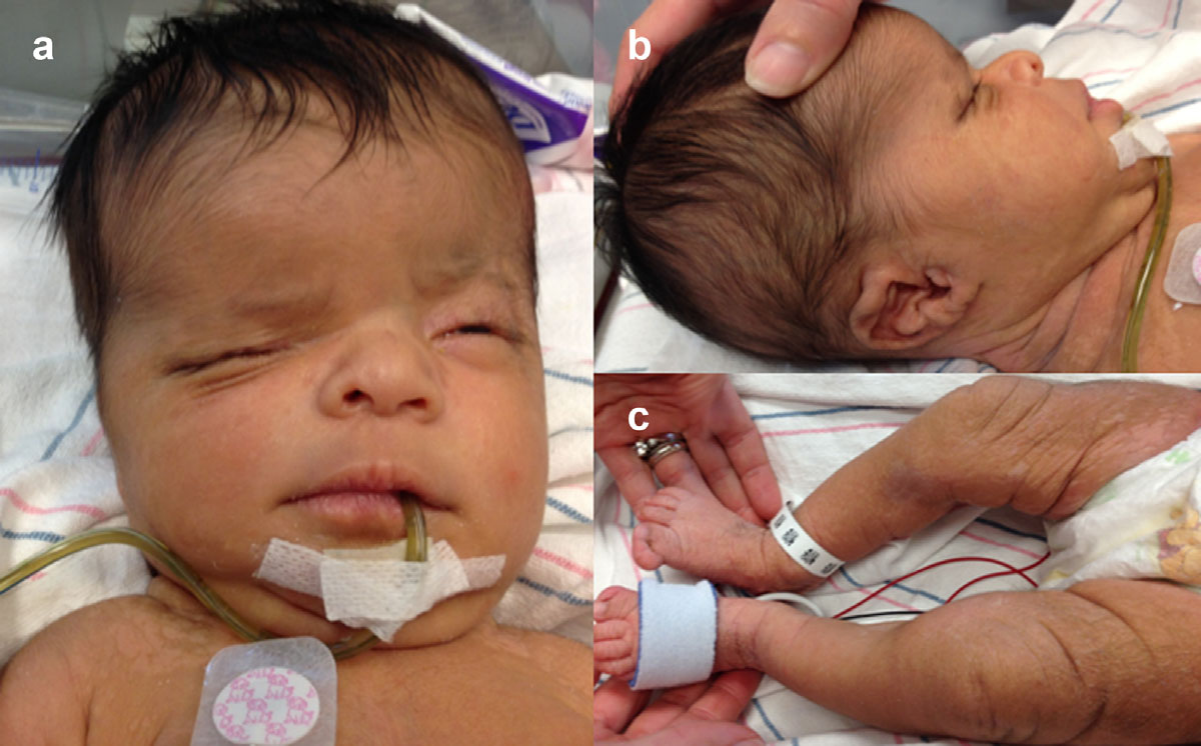
(a–c) Craniofacial, extremity, and cutaneous features in a newborn with Curry–Jones syndrome. Notable features include asymmetric frontal bossing, hypoplastic left supraorbital ridge, right orbital dystopia and downward displacement of right palpebral fissure, low nasal bridge, short nose with bulbous nasal tip. Profile of craniofacial features highlights a small right ear with irregular helical contour and redundant tissue. Bilateral pre‐axial polydactyly involving the great toe of right and left lower extremities. Patchy skin distribution with areas of pigmentation and depigmentation and atrophic texture. [Color figure can be viewed at http://wileyonlinelibrary.com]

The proband was noted to have multiple dysmorphic features at birth (Figure 1). She had macrocephaly, fusion of the left coronal suture, facial asymmetry with a hypoplastic left supra‐orbital ridge and contralateral frontal bossing, and flat occiput. The nasal bridge was depressed with short, anteverted nares and the bulbous nasal tip deviated to the right. The right ear was smaller than the left with an irregular contour and redundant tissue. Ocular anomalies included hypertelorism with irregular contour of the eyelids, microphthalmia, and inability to open the right eye spontaneously. Ectopic eyelashes were noted on the upper eyelid. Dilated fundoscopic exam revealed sectoral (partial) clouding of both corneas, right greater than left. The palate was intact and the mandible was normal. There was pre‐axial polydactyly with bilateral partial duplication of the thumbs and great toes. A single transverse palmar crease was present on the right hand. Examination of the skin revealed thickened, marbled areas normally pigmented and hypopigmented skin on the right trunk, and bilaterally on the lower extremities (see right shoulder in Figure 1a and lower extremities in Figure 1c). The skin had an atrophic, pebbly texture with patches of abnormal hair on the extremities. The abdomen was distended but soft without palpable masses or organomegaly. The remainder of the examination including respiratory, cardiovascular, genitourinary, and neurologic systems was unremarkable. Upon recognition, the distinctive pattern of cutaneous findings, craniosynostosis, and pre‐axial polysyndactyly by an examiner (MCJ) with previous experience with CJS, a clinical diagnosis of CJS was made in the proband.

Renal ultrasound revealed mild bilateral hydronephrosis and cranial ultrasound suggested callosal agenesis. Subsequent brain MRI revealed near‐complete agenesis of the corpus callosum. Radiographs of the upper extremities showed pre‐axial polydactyly with osseus syndactyly of the extra digit to the thumb and a short first metacarpal on the right hand; the left hand also had pre‐axial polysyndactyly. Radiographs of the lower extremities showed bilateral pre‐axial polydactyly. CT scan of the head showed left coronal craniosynostosis as well as multiple intrasutural bones being noted, similar to wormian bones. A SNP‐oligonucleotide microarray showed 46, XX without pathogenic copy number variation.

On the fourth day of life, the patient was evaluated for decreased oral intake followed by 1 day of bilious emesis and abdominal distention. Upright abdominal radiographs revealed significant proximal bowel dilatation. She was decompressed with oral‐gastric (OG) suction and bowel rest with initial improvement in symptoms. However, she continued to have bilious OG output in the absence of bowel movements for multiple days. Serial abdominal radiographs did not show obvious obstruction, although she had very delayed transit of contrast through her bowel and rectum. Rectal biopsies were obtained which revealed the presence of ganglion cells, effectively ruling out Hirschsprung disease. Rare hypertrophic nerve bundles were also noted on biopsy. After rectal biopsy, an upper GI radiographic series with barium contrast revealed malrotation without volvulus and delayed emptying of contrast through the cecum and rectum.

**Figure 2:**
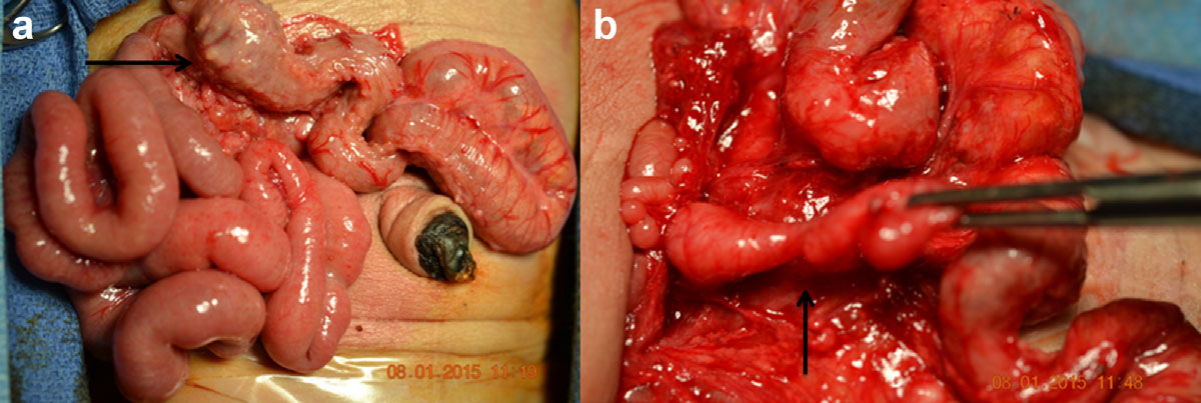
(a and b) Malrotation and small discrete nodules adherent to the small and large bowel. Congenital intestinal duplication originating posterior to the pancreas. [Color figure can be viewed at http://wileyonlinelibrary.com]

**Figure 3:**
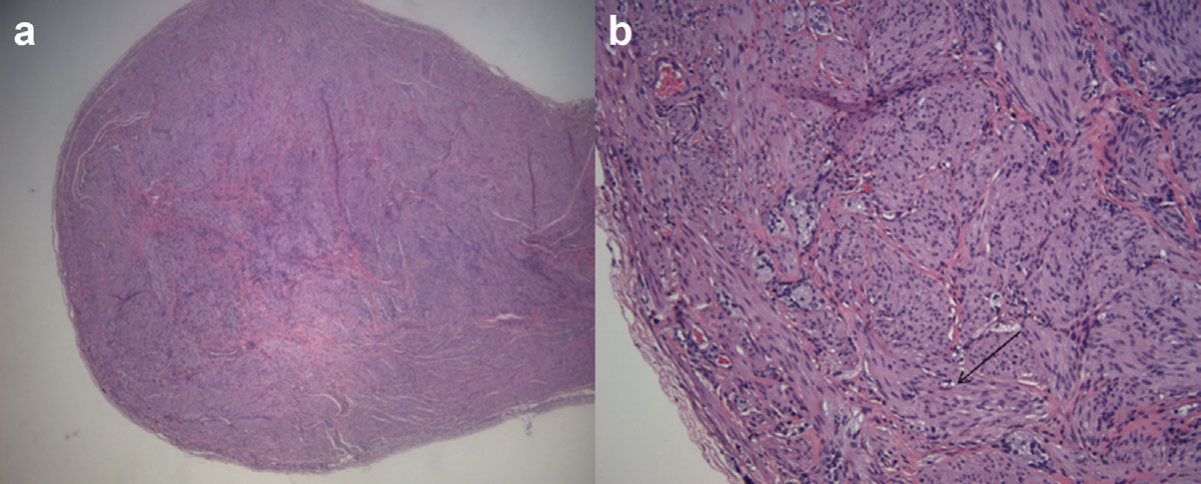
(a and b) H&E staining of discrete mass from the cecal mesentery. Appearance at 200× magnification reveals intermyenteric ganglion cells embedded in smooth muscle. Arrow indicates smooth muscle fibers. [Color figure can be viewed at http://wileyonlinelibrary.com]

On day 14, the infant underwent exploratory laparotomy and Ladd procedure to address the malrotation. During laparotomy, the patient was found to have a curvature at the duodenum that appeared almost twisted but no Ladd bands crossing the small bowel, and no obvious point of obstruction. Throughout the abdomen on the mesentery and the surface of the bowel and appendix were small tumors (Figure 2a). Formalin‐fixed biopsies of these tumors revealed smooth muscle bundles with intermyenteric ganglion cell nests (Figure 3), a distinctive feature that provided further confirmation of the diagnosis of CJS. Additionally, the patient had a blind‐ended tubular structure that appeared to originate from behind the pancreas with increasing diameter (1.5 cm) as it descended posteriorly, consistent in appearance with a congenital intestinal duplication (Figure 2b). The postoperative course was complicated by prolonged ileus and required parental nutrition until the 27th day of life. The infant was discharged home on full oral feeding after 45 hospital days.

Following NICU discharge, the infant had intermittent emesis, poor weight gain on 24 kcal fortified formula, and chronic constipation. She was admitted at 5 months of age for failure to thrive. The infant was noted to have a focal area of esophageal narrowing just below the level of the thoracic inlet on a modified barium swallow study. An upper GI and small bowel series with barium contrast demonstrated diffuse intestinal distension. Anorectal manometry revealed borderline elevated resting pressure of the internal anal sphincter with normal recto‐ano inhibitory reflex. The esophageal pH study was normal. A follow‐up brain MRI demonstrated left cerebellar tonsillar ectopia, near complete agenesis of the corpus callosum, colpocephaly, increased size of the lateral and third ventricles, and a dysgenesis of the tectal plate and delayed myelination for age. The infant underwent gastrostomy tube placement for dysmotility and poor weight gain. Intraoperative findings included a markedly dilated transverse colon. The infant was discharged home on continuous gastrostomy tube feedings after 45 hospital days.

The infant was readmitted when she was 8 months old for feeding intolerance and weight loss. Upon admission, this formerly macrosomic infant had a weight for age less than the first percentile (*z* = −3.32), length was less than the first percentile (z = −2.35), and head circumference at the 73rd percentile (z = 0.63) on WHO growth charts. She developed intermittent abdominal distension, emesis, and irritability when gastrostomy feedings were advanced. A CT of the abdomen and pelvis demonstrated diffuse colon dilatation without obvious mechanical obstruction. The abdominal distension improved with placement of a nasogastric tube. Based on the imaging findings, the diagnosis of CJS, and known anatomical abnormalities from prior procedures, the clinical picture was most likely to be caused by colonic pseudo‐obstruction. An ileostomy was recommended to manage colonic pseudo‐obstruction. However, after multiple meetings with the family the parents declined the ileostomy. The infant was discharged home with the family at 9 months on hospice care and subsequently died.

## 3 MOLECULAR STUDIES

**Figure 4:**
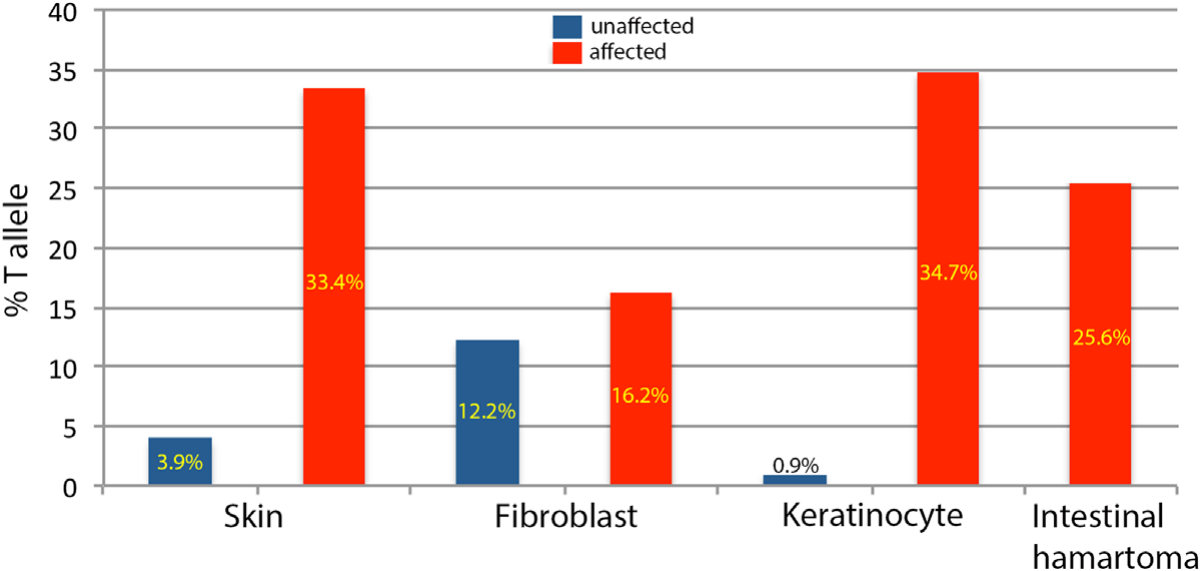
Distribution of *SMO* c.1234C>T, p.Leu412Phe mutant T alleles in affected (red bars) and clinically unaffected (blue bars) tissues. Dermis and epidermis layers are represented by cultured fibroblasts and keratinocytes, respectively. [Color figure can be viewed at http://wileyonlinelibrary.com]

Informed consent was obtained with an IRB‐approved consent form prior to participation in research. For analysis of *SMO*, we collected normal and affected skin samples as well as formalin‐fixed paraffin‐embedded (FFPE) sections of the intestinal smooth muscle hamartoma. Skin samples were placed in tissue culture dishes to isolate fibroblasts and keratinocytes. After 2 days of culture, fibroblasts were removed by trypsin digestion (and subsequently passaged twice before analysis), while the keratinocytes (which surround the skin sample) were harvested using a cell scraper. DNA was extracted from skin (including isolated fibroblasts and keratinocytes) by Proteinase K digestion followed by phenol/chloroform extraction and ethanol precipitation, while DNA from FFPE material (five 10 μM sections) was isolated using the QIAamp DNA FFPE Tissue kit (Qiagen). Cellular identities of keratinocytes and fibroblasts were confirmed with RT‐PCR analysis of expression of the two distinguishing *FGFR2* isoforms (IIIb is only expressed in keratinocytes while IIIc is only expressed in fibroblasts, data not shown). The same mutant allele of *SMO* (c.1234C>T, p.Leu412Phe) found in other cases of CJS (Twigg et al., 2016) was identified. Figure 4 presents the distribution of mutant and wild‐type allele for affected and unaffected skin samples. The mutant allele was found in both affected dermis (represented by fibroblasts; T allele frequency 16.2%) and at a higher level in affected epidermis (represented by keratinocytes; T allele frequency 34.7%). The mutant T allele was present also in unaffected dermis and epidermis but at a lower level compared to affected skin. In the intestinal hamartoma the mutant allele was present in 25.6% of the sample.

## 4 DISCUSSION

Important insights into the clinical and molecular pathogenesis of CJS can be drawn from the present case. From a molecular standpoint, a recurrent mosaic mutation in *SMO* results in malformations of the gastrointestinal tract and skin. Analyses of affected skin and intestinal smooth muscle hamartoma show an increased frequency of the mutant *SMO* allele compared with unaffected tissues. In all affected tissues studied, the mutant allele is present at a frequency less than 50%, which reflects tissue mosaicism. Twigg et al. (2016) recently identified a mosaic gain of function mutation in *SMO* (c.1234C>T, p.Leu412Phe) in seven other cases with CJS in multiple affected tissues including skin, FFPE samples of medulloblastoma, intestinal myofibromas, or smooth muscle hamartomas. Within the skin, the mutant allele frequency varied with the phenotypic findings of the skin (hyperpigmented or affected compared with unaffected) but also with the layers of the skin. The factors that drive a higher mutant allele frequency within the epidermis are unknown. In addition, detection of the mutant allele within apparently unaffected skin at a low level suggests that the mutation arose early in skin development. Indeed, the presence of mosaicism as well as detection of the mutant allele in multiple different tissue types suggests that the mutation occurred as a post‐zygotic error early in embryonic development.

*SMO* is a key component of the HH‐GLI pathway and its protein product, smoothened, is essential for normal HH signal transduction. The HH‐GLI pathway has widespread roles in embryonic development (Briscoe & Thérond, 2013). Specifically, within the gastrointestinal system, prior studies have demonstrated the role of the HH‐GLI pathway for cellular differentiation, tissue patterning, and axis determination. (Hirokawa, Tanaka, Okada, & Takeda, 2006; Ramalho‐Santos, Melton, & McMahon, 2000; Shook & Keller, 2003). Congenital gastrointestinal malformations including malrotation have been reported in studies of *Shh* ^−/−^ mice (Ramalho‐Santos et al., 2000). Similar studies have not been replicated in *Smo* ^−/−^ mice as the knockout results in an embryonic lethal phenotype (Zhang, Ramalho‐Santos, & McMahon, 2001).

The identical recurrent mutation in *SMO* that accounts for the multiple malformations in CJS is also observed in several cancers including ameloblastoma (Sweeney et al., 2014), basal cell carcinoma (Atwood et al., 2015; Sharpe et al., 2015; Xie et al., 1998), medulloblastoma (Jones et al., 2012; Pugh et al., 2012), and meningioma (Brastianos et al., 2013). The mutant protein results in constitutive activation of smoothened in the absence of HH signaling in mouse *Smo* ^−/−^ embryonic fibroblasts and is associated with increased cell division (Sweeney et al., 2014). This finding may help account for the propensity to develop benign neoplasms such as smooth muscle hamartomas in affected tissues in the bowel.

The mutation in *SMO* leads to constitutive activation of HH signaling, hence disrupting the HH pathway in embryonic gastrointestinal development, resulting in gastrointestinal malformations and impaired peristalsis. The gastrointestinal dysmotility may be due to aberrant enteric nervous system development in the context of structural gastrointestinal malformations. The severity of the phenotype likely reflects the amount and distribution of the mutant *SMO* allele in affected tissues along the gastrointestinal tract.

Clinically, gastrointestinal abnormalities are common in CJS and are a major cause of morbidity in this condition. Table 1 provides comparison of gastrointestinal features in CJS. In children with known gastrointestinal abnormalities, onset of symptoms occurred in the neonatal period in three other cases in addition to the present case. Gastrointestinal manifestations included structural malformations such as malrotation (*n* = 5), congenital short gut (*n* = 1), and intestinal duplication (*n* = 1). One child underwent screening imaging at 9 months of age due to reports of gastrointestinal abnormalities in CJS and was found to have an asymptomatic malrotation (Grange et al., 2008). These findings highlight the importance of screening abdominal imaging for gastrointestinal malformations in children with suspected CJS.

**Table 1:** Gastrointestinal features in Curry–Jones syndrome

Patient	Case	Gender	Gastrointestinal features^a^	Onset^b^	Surgery^c^	GI myofibromas or smooth muscle hamartomas	Recurrence^d^
Temple et al. (1995) #1	1	M	**Recurrent pseudo‐obstruction** requiring colostomy, anal stenosis	5 months	+	Large bowel	+
Temple et al. (1995) #2	2	F	No symptoms	None	−	Unknown	−
Temple et al. (1995) #3	3	M	**Esophageal dysmotility**	None	−	Unknown	−
Temple et al. (1995) #4	4	F	**Constipation**; recurrent massive GI bleeding of unclear etiology at 10 years	4 months	+	Mesentery	+
Temple et al. (1995) #5	5	M	**Malrotation**; congenital short gut; obstruction due to adhesions; severe GERD; dysmotility of small intestine; jejunostomy enteral feeding	8 days	+	Mesentery	+
Mingarelli et al. (1999)	6	M	No symptoms	None	−	Unknown	−
Thomas et al. (2006)	7	M	No symptoms	None	−	Unknown	−
Grange et al. (2008) #1	8	M	**Asymptomatic malrotation** identified on imaging at 9 months; persistent abdominal distension	Not reported	+	Large bowel, mesentery, retroperitoneum	+
Grange et al. (2008) #2	9	M	**Malrotation**; GERD; pseudo‐obstruction; chronic constipation; obstipation and volvulus requiring sub‐total colectomy	<30 days	+	Large bowel	+
Twigg et al. (2016) #8	10	F	**Malrotation**; diarrhea; malabsorption; pseudo‐obstruction	<30 days	+	Small bowel and appendix, mesentery	+
Present case (Twigg #10)	11	F	**Malrotation**; intestinal duplication; pseudo‐obstruction; chronic constipation; gastrostomy enteral feeding	4 days	+	Small and large bowel, mesentery	+

^a^ Presenting gastrointestinal feature is bolded.

^b^ Age of onset of gastrointestinal symptoms.

^c^ Abdominal surgery for malrotation, pseudo‐obstruction, or gastrointestinal bleeding.

^d^ Recurrent gastrointestinal symptoms.

Symptoms of underlying gastrointestinal pathology may have delayed presentation in children with CJS. Beginning at 10 years of age, one child with CJS had recurrent episodes of bloody stools and orthostasis requiring hospitalization and multiple blood transfusions (noted in the table in Grange et al., 2008). During the first episode of lower gastrointestinal bleeding, she had an exploratory laparotomy with resection of a Meckel's diverticulum. Mesenteric smooth muscle hamartomas were noted and jejunal biopsy revealed lymphangiectasia. Another child underwent subtotal colectomy for severe constipation and volvulus at 10 years of age. Gastrointestinal smooth muscle hamartomas and benign colonic polyps were noted in the resected specimen (C. Clericuzio, personal communication, February 6, 2016.). Motility disorders have been observed and have ranged in severity from chronic constipation (*n* = 3), small intestine dysmotility (*n* = 1), to severe pseudo‐obstruction (*n* = 4), requiring multiple hospitalizations and colostomy. Children affected with pseudo‐obstruction often required multiple hospitalizations for symptomatic management and nutritional support and one child underwent colostomy. Despite surgical intervention, children with CJS and gastrointestinal abnormalities tend to have recurrent abdominal symptoms and poor postnatal growth. The prognosis of gastrointestinal features is guarded as symptoms often recur or persist despite surgical intervention. Vigilant and coordinated inter‐disciplinary care with medical, nutritional, and surgical teams is crucial to mitigate the morbidity due to gastrointestinal abnormalities and optimize postnatal growth.

All cases of CJS in the literature have a distinctive pattern of malformation allowing clinical diagnosis of this condition. Individuals newly diagnosed with CJS should have a thorough evaluation to characterize the degree of gastrointestinal manifestations (see Table 2 for recommended evaluation). This condition probably reflects a high level of mosaicism in multiple affected tissues. Individuals with lower level mosaicism may have less obvious features, particularly cutaneous features (Khamaysi et al., 2016). The diagnosis of CJS should be considered in infants with craniosynostosis, polysyndactyly, or patchy skin manifestations, who have unexplained recurrent gastrointestinal symptoms.

**Table 2:** Suggested initial evaluations and treatment following a diagnosis of CJS

Feature	Evaluation
Gastrointestinal malformations	Contrast study to exclude intestinal malrotation MRI abdomen/pelvis
Delayed passage of meconium or constipation	Barium enema Rectal suction biopsy Consider anorectal manometry
Abdominal distension or feeding intolerance	Three view abdominal radiographs to assess for obstruction, nasogastric tube decompression Consultation with pediatric surgeon Caregiver education on recognition of early symptoms/signs of bowel obstruction
Risk for malnutrition	Evaluation for anatomic malformations, dysmotility, and pseudo‐obstruction as above Early nutrition consult
Gastrointestinal bleeding	Nasogastric saline lavage and aspirate Stool guiac studies Consider upper endoscopy and/or colonoscopy Consider technetium‐99m (99mTc) scan for Meckel's diverticulum
